# Upcycling Used Tyre Rubber into Carbon Adsorbents: Effect of Physical Activation on Porous Structure and Comparative Evaluation for *p*-Chlorophenol Removal

**DOI:** 10.3390/polym18202544

**Published:** 2026-10-16

**Authors:** César Troca-Torrado, María Francisca Alexandre-Franco, Juan Manuel Garrido-Zoido, Eduardo Manuel Cuerda-Correa, Vicente Gómez-Serrano

**Affiliations:** Department of Organic and Inorganic Chemistry, Faculty of Science, University of Extremadura, 06006 Badajoz, Spain; cesar_trocato@hotmail.com (C.T.-T.); emcc@unex.es (E.M.C.-C.); vgomez@unex.es (V.G.-S.)

**Keywords:** used tyre rubber, carbonaceous adsorbents, physical activation, steam activation, porous structure, processing–structure–property relationship, *p*-chlorophenol, adsorption, circular economy, polymeric waste

## Abstract

The porous architecture of carbons derived from polymeric waste is largely governed by the physical activation atmosphere. In this work, used tyre rubber (UTR) was converted into carbonaceous adsorbents by pyrolysis followed by physical activation in air, CO_2_ or steam. The influence of the activation route on porous structure, surface chemistry and adsorption performance was systematically investigated. The materials were characterised by N_2_ adsorption at −196 °C, mercury intrusion porosimetry, scanning electron microscopy (SEM), FT-IR spectroscopy and point-of-zero-charge (pH_pzc_) measurements. The resulting materials were evaluated for the removal of *p*-chlorophenol from aqueous solution and comparatively assessed against three commercial carbon materials. Steam activation proved to be the most effective treatment, increasing the BET surface area from 64 to 344 m^2^ g^−1^ and the micropore volume from 0.05 to 0.23 cm^3^ g^−1^ while generating a hierarchical micro-/mesoporous structure that significantly enhanced pore accessibility and mass transport. Among the UTR-derived carbons, the steam-activated sample exhibited the highest equilibrium *p-C*p uptake and adsorption kinetics approaching those of a commercial activated carbon despite its lower specific surface area. Kinetic and equilibrium analyses demonstrated that adsorption performance depends not only on the extent of microporosity but also on pore accessibility and network connectivity, highlighting the critical role of transport mesoporosity in facilitating molecular diffusion towards adsorption sites. The adsorption results are consistent with an important contribution from accessible microporosity, while π–π interactions between *p*-chlorophenol and aromatic carbon domains may also contribute. These findings demonstrate that tailoring porous structure through physical activation provides an effective strategy for transforming UTR into high-performance carbon adsorbents and establishes robust processing–structure–property relationships that can guide the rational design of waste-derived carbon adsorbents.

## 1. Introduction

The transition towards a circular economy has stimulated the development of innovative strategies for transforming end-of-life polymeric wastes into high-value functional materials [1,2,3,4,5]. Rather than being regarded solely as an environmental burden, these waste streams are increasingly recognised as valuable secondary carbon resources that can be converted into functional carbon materials for applications in adsorption, catalysis, electrochemical energy storage and sensing [4,5,6,7,8,9]. Among these valorisation routes, the production of carbonaceous adsorbents has attracted particular attention because it simultaneously addresses the management of difficult-to-recycle polymeric wastes while responding to the growing demand for effective materials for environmental remediation [4,5,10,11,12,13]. Consequently, the rational design of carbonaceous adsorbents derived from polymeric waste has become an active research area within the broader framework of sustainable materials science and circular economy technologies [2,3,7,8,14].

Among the wide variety of polymeric wastes currently under investigation, used tyre rubber (UTR) has emerged as one of the most attractive carbon precursors because of its abundance, high carbon content and unique chemical composition. Approximately one-third of the tyre mass consists of carbon black, whose carbon content reaches about 70–75 wt.%, making UTR an exceptionally rich secondary carbon resource for the production of porous carbon materials [14,15,16]. Tyre rubber consists primarily of natural and synthetic elastomers reinforced with carbon black and inorganic additives, forming a highly crosslinked material that is resistant to biological degradation and difficult to recycle through conventional technologies [1,6,17]. Although mechanical recycling, energy recovery and other valorisation strategies continue to represent the principal management routes for this waste stream [6,7,8,18,19,20,21,22,23,24], their ability to exploit its intrinsic carbon value and functional potential remains limited [6,7,8,16,17]. In contrast, thermochemical conversion through pyrolysis followed by physical activation has emerged as one of the most promising approaches for transforming UTR into high-value porous carbon materials [10,11,12,15,25,26,27,28]. Since carbon black already provides a highly aromatic carbon framework, the primary role of physical activation is to develop its internal porosity and accessible surface area, thereby converting the pyrolytic char into an efficient carbon adsorbent. Besides contributing to waste minimisation, this approach provides a route for valorising the carbon content of UTR into adsorbents with tunable textural properties that can be benchmarked against conventional commercial activated carbon in environmental remediation applications [10,11,12,13,15,16,26,28,29].

Among the different applications of these porous carbon materials, environmental remediation—and particularly water purification—has become one of the most active research areas owing to the increasing demand for efficient technologies capable of removing hazardous organic contaminants from water. Natural self-purification processes are only capable of degrading or eliminating pollutants when contaminant loads remain low relative to the available water resources. However, rapid industrialisation and the continuously increasing demand for manufactured products have led to the large-scale release of organic contaminants into aquatic environments, making water pollution one of the major environmental challenges worldwide. Among these contaminants, chlorinated organic compounds are of particular concern because, in addition to being discharged through numerous industrial activities [30,31], some are also generated as undesirable by-products during drinking water chlorination processes [32,33].

Within this group, chlorophenols have attracted considerable attention owing to their widespread use as intermediates in the manufacture of pesticides, herbicides, disinfectants, dyes, pharmaceuticals and wood preservatives [30,34]. Their continuous release into aquatic environments, together with their high toxicity, environmental persistence, limited biodegradability and bioaccumulation potential, has led to their classification as priority organic pollutants by environmental protection agencies [30,34]. Besides imparting unpleasant taste and odour to water, chlorophenols have been associated with mutagenic and carcinogenic effects, DNA damage, chromosomal aberrations, immunotoxicity, histopathological alterations and adverse effects on the nervous and hepatic systems, highlighting the urgent need for effective technologies capable of removing these contaminants from polluted waters [30,35,36,37,38,39,40,41]. Among chlorophenols, *p*-chlorophenol (*p-C*p) has become one of the most widely employed model compounds for evaluating carbonaceous adsorbents because its physicochemical properties are representative of a broad family of chlorinated phenolic compounds commonly detected in industrial wastewaters [30,34,42,43,44,45].

The environmental significance of chlorophenols has stimulated the development of numerous technologies for their removal from contaminated waters, including biodegradation, electrochemical oxidation, sonochemical degradation, photocatalysis and adsorption [30,43,46,47]. Among these approaches, adsorption remains one of the most efficient technologies owing to its operational simplicity, high removal efficiency and relatively low energy requirements [13,43,44,45,48,49,50,51]. Commercial activated carbons continue to represent the benchmark adsorbents because of their high specific surface area, well-developed microporosity and favourable surface chemistry, which together provide excellent adsorption capacities and rapid adsorption kinetics for a wide range of organic pollutants [13,45,48,51,52,53]. Nevertheless, their production generally relies on non-renewable raw materials and energy-intensive manufacturing processes, increasing both production costs and environmental impacts [13,45,51]. Consequently, considerable research efforts have been devoted to identifying abundant waste-derived alternative precursors capable of producing high-performance carbon adsorbents through efficient valorisation routes. In this regard, UTR represents an especially attractive precursor because of its high carbon content—largely associated with its carbon black fraction [14,15,16]—and its ability to develop a highly porous structure through pyrolysis followed by physical activation using air, CO_2_ or steam [25,26,27,28,29,54]. This strategy therefore provides a route for valorising UTR as a precursor for carbon adsorbents intended for water-treatment applications.

Although UTR has been widely investigated as a precursor for carbonaceous adsorbents, previous studies have generally focused on optimising a single activation route or reporting adsorption performance for a particular pollutant. Comparatively fewer studies have examined, under a common experimental framework, how different physical activation atmospheres modify the complete pore hierarchy of tyre-derived carbons and how these changes control both equilibrium capacity and adsorption kinetics. In particular, the respective roles of adsorption microporosity and transport meso-/macroporosity remain difficult to separate when only BET surface area or total pore volume is considered.

The distinctive contribution of the present work is therefore the direct comparison of air, CO_2_ and steam activation applied to UTR-derived chars, combined with complementary N_2_ adsorption and mercury intrusion porosimetry and benchmarking against three commercial carbon materials with markedly different pore architectures. This approach enables the adsorption response to be interpreted in terms of a processing–structure–property relationship rather than through BET surface area alone, with particular emphasis on the balance between micropore development, pore accessibility and transport connectivity.

Based on these considerations, the present work investigates how different physical activation atmospheres (air, CO_2_ and steam) govern the development of the porous structure of used tyre-derived carbons and how these structural modifications influence the adsorption of *p*-chlorophenol from aqueous solutions. The prepared materials were comprehensively characterised by N_2_ adsorption at −196 °C, mercury intrusion porosimetry, FT-IR spectroscopy and pH_pzc_ measurements, and their performance was benchmarked against three commercial carbon materials. Adsorption behaviour was evaluated through kinetic and equilibrium studies to establish the processing–structure–property relationships governing the design of efficient carbon adsorbents derived from polymeric waste.

## 2. Materials and Methods

### 2.1. Materials

Used tyre rubber (UTR) was kindly supplied by the Higher Polytechnic School of Alcoy (Alicante, Spain). As received, the material had already been freed from steel wires, shredded, cryogenically size-reduced, and sieved. After these conditioning steps, the particle size ranged from 1 to 3 mm. The elemental composition of UTR was as follows: C, 83.52 wt.%; H, 7.28 wt.%; N, 0.33 wt.%; S, 0.02 wt.%; O, 3.08 wt.% [15]; ash content, 7.0 wt.%. The commercial carbon materials used for comparison were Darco 20–40 activated carbon (D, Sigma Aldrich, Madrid, Spain), Vulcan 6 carbon black (V6, Cabot S.A., Leioa, Spain), and A20 carbon fibre (A20, supplied by Osaka Gas through the University of Alicante, Spain). *p*-Chlorophenol (*p-C*p, purity > 99%) was purchased from Panreac Química S.A.U. (Castellar del Vallès, Spain).

### 2.2. Preparation of Activated Carbons, ACs

UTR-derived carbonaceous adsorbents were prepared by physical activation in two successive stages: pyrolysis under N_2_ followed by activation in air, CO_2_, or steam. First, approximately 10 g of UTR was heat-treated in a vertical cylindrical furnace (CHESA, Spain) equipped with a stainless-steel reactor under a nitrogen flow of 100 mL min^−1^. Subsequently, about 5 g of the resulting pyrolysed product was partially gasified either in air or CO_2_ (100 mL min^−1^) in a horizontal furnace (Carbolite GHA 12/450, supplied by Verder Scientific Iberia, Eibar, Spain), or in steam using two closely connected horizontal furnaces arranged in series for steam generation and char activation [13]. During both carbonisation and activation, the heating rate from room temperature to the maximum heat-treatment temperature (MHTT) was 10 °C min^−1^, and the final temperature was held for 2 h. After cooling to room temperature, the resulting solids were weighed and stored in airtight plastic containers. The preparation conditions, yields, and sample codes are summarised in Table 1.

### 2.3. Characterisation of Carbonaceous Adsorbents

#### 2.3.1. Textural Characterisation

The textural properties of the samples were characterised by N_2_ adsorption at −196 °C and mercury porosimetry. N_2_ adsorption isotherms were obtained using a Quantachrome Autosorb-1 semi-automatic analyser. Before the measurements, the samples were oven-dried overnight at 120 °C. Approximately 0.15 g of each sample was then placed in a glass sample holder and degassed in the adsorption apparatus at 250 °C for 12 h under a pressure below 10^−3^ Torr. The specific surface area was calculated using the Brunauer–Emmett–Teller (BET) equation [55], and the micropore volume was estimated by the Dubinin–Radushkevich (D-R) equation [56]. Mercury intrusion measurements were carried out using a Quantachrome Autoscan mercury porosimeter with approximately 0.3 g of sample. Scanning electron microscopy (SEM) was also used to examine the surface morphology and verify the presence of porosity.

#### 2.3.2. Surface Chemistry

The surface chemistry of UTR, the prepared adsorbents, and the commercial carbon materials were analysed by FT-IR spectroscopy and determination of the pH at the point of zero charge (pH_pzc_) [57]. FT-IR spectra were recorded on a Perkin–Elmer 1720 spectrometer in the range 4000–400 cm^−1^, using 50 scans at a resolution of 2 cm^−1^. KBr pellets were prepared by thoroughly mixing the sample with KBr in a 1:900 weight ratio in an agate mortar. The resulting mixture was pressed in a Perkin–Elmer manual hydraulic press at 10 tons for 3 min. A KBr pellet containing the same amount of dispersant was used as the background.

The pH_pzc_ was determined using 0.01 M NaCl aqueous solutions adjusted to initial pH values of 2, 4, 5, 7, 9, and 11 with 0.1 M HCl or NaOH. In each experiment, 5 mL of solution was brought into contact with 0.015 g of sample in a set of test tubes, and the suspensions were kept under continuous stirring for 48 h. The supernatant was then separated by filtration, and its final pH was measured. The pH_pzc_ value was obtained from the plot of initial pH versus final pH.

### 2.4. Adsorption from Solution

#### 2.4.1. Adsorbate

*p*-Chlorophenol (C_6_H_5_OCl, purity > 99%) purchased from Panreac Chemical S.A. was used as the single adsorbate in this study.

#### 2.4.2. Adsorption Studies

Adsorption experiments were carried out by the batch method using a Selecta thermostatic shaker bath (Unitronic-ORC) at 25 °C and 50 oscillations min^−1^. In a typical kinetic experiment, approximately 0.1 g of adsorbent was added to 25 mL of a 10^−3^ mol L^−1^ aqueous *p-C*p solution in 25 mL glass test tubes fitted with Bakelite screw caps. Contact times ranged from 5 min to several days. Equilibrium data were obtained by varying the adsorbent mass from 0.002 to 0.300 g while keeping the solution volume and initial *p-C*p concentration constant. In all cases, the residual *p-C*p concentration in solution was determined by UV–Vis spectrophotometry using a Shimadzu UV-3101PC instrument. Before the adsorption measurements, the stability of *p-C*p in aqueous solution was evaluated at different concentrations and pH values by monitoring the absorbance as a function of time. Under these conditions, *p-C*p was found to remain stable in aqueous solution for up to 10 days. Absorbance was measured at 279.5 nm. The initial pH of the 10^−3^ mol L^−1^ *p-C*p solutions used in the adsorption experiments was approximately 5.0 and was not externally adjusted.

All adsorption experiments were conducted at 25 °C under the common batch conditions described above so that the relative performance of the different carbon materials could be directly compared. The kinetic experiments were performed by varying contact time at fixed adsorbent mass and initial *p-C*p concentration, whereas equilibrium isotherms were obtained by varying adsorbent mass while maintaining the initial solution volume and *p-C*p concentration constant. The residual solution concentration determined by UV–Vis spectroscopy was used to calculate the adsorbed amount by mass balance.

The amount of *p-C*p adsorbed at equilibrium was calculated by the following mass-balance equation:

(1) *q_e_* = (*C*_0_ − *C_e_*) · *V*/*W*

where *q_e_* is the amount of *p-C*p adsorbed per unit mass of adsorbent (mol g^−1^), *C_0_* and *C_e_* are the initial and equilibrium *p-C*p concentrations in solution (mol L^−1^), respectively, *V* is the solution volume (L) and *W* is the mass of adsorbent (g).

#### 2.4.3. Kinetic and Equilibrium Modelling

The kinetic data were analysed using the pseudo-first-order and pseudo-second-order models [58,59], respectively:

(2) $$\log\left(q_{e}-q_{t}\right)=\log q_{e}-\frac{k_{1}}{2.303}t$$

(3) $$\frac{t}{q_{t}}=\frac{1}{q_{e}^{2}k_{2}}+\frac{t}{q_{e}}$$

where *k*_1_ (h^−1^) and *k*_2_ (g mol^−1^ h^−1^) are the pseudo-first-order and pseudo-second-order rate constants, respectively, and *q_e_* is the equilibrium amount of *p-C*p adsorbed (mol g^−1^). The equilibrium data were analysed using the Langmuir [60], Freundlich [61], Sips [62], and Redlich-Peterson equations [63]:

(4) q_e_ = (Q_m_ b C_e_)/(1 + b C_e_)

(5) q_e_ = K_F_ C_e_ ^(1/n)^

(6) $$q_{e}=\frac{q_{s}(K_{S}\cdot C_{e}{)}^{n_{S}}}{1+(K_{S}\cdot C_{e}{)}^{n_{S}}}$$

(7) $$q_{e}=\frac{K_{R}\cdot C_{e}}{1+a_{R}\cdot C_{e}^{\beta }}$$

where *Q_m_* is the Langmuir saturation capacity, *b* is the Langmuir affinity constant, *K_F_* and 1/*n* are the Freundlich constant and exponent, respectively, *q_S_*, *K*_S_ and *n_S_* are the Sips parameters, and K_R_, *a_R_* and *β* are the Redlich–Peterson parameters.

For all four equilibrium models, the parameters were estimated by unweighted nonlinear least-squares regression of the untransformed *q_e_* vs. *C_e_* data by minimising the sum of squared errors (SSE); no linearised forms were used. Goodness of fit was compared using R^2^, adjusted R^2^, RMSE and the corrected Akaike information criterion (*AICc*). The statistics were calculated as

(8) $$R^{2}=1-\frac{\mathrm{SSE}}{\mathrm{SST}}$$

(9) $$\mathrm{Adjusted}\;R^{2}=\frac{1-\left(1-R^{2}\right)(N-1)}{\left(N-P-1\right)}$$

(10) $$\mathrm{RMSE}={\left[\frac{\mathrm{SSE}}{\left(N-p\right)}\right]}^{1/2}$$

and

(11) $${\mathrm{AIC}}_{c}=\frac{N\cdot \ln\left(\frac{\mathrm{SSE}}{N}\right)+2p+2p(p+1)}{\left(N-p-1\right)}$$

where *N* is the number of experimental points and *p* is the number of adjustable parameters. The isotherm equations were used as empirical comparative models and were not interpreted as direct proof of a unique adsorption mechanism; fitted saturation or affinity parameters were not assigned physical significance when the experimental data did not define a saturation plateau.

## 3. Results and Discussion

### 3.1. Preparation of the Activated Carbons

#### 3.1.1. Yields

The carbonisation yields were 36.6 wt.% for H600 and 33.0 wt.% for H900 (Table 1). The modest decrease upon increasing the maximum heat-treatment temperature from 600 to 900 °C indicates that most devolatilisation had already occurred by 600 °C, consistent with the reported thermal decomposition behaviour of UTR [16,27,64,65]. These values also agree with carbonisation yields commonly reported for tyre-derived chars under inert atmospheres [15,52,54]. The relatively high solid yields reflect the high carbon-black content of UTR and confirm its suitability as a carbon-rich precursor [66,67].

#### 3.1.2. Physical Activation

The activation yields obtained for the prepared activated carbons are summarised in Table 1. The process yield reached 95 wt.% for air activation, 88 wt.% for CO_2_ activation and 54 wt.% for steam activation, following the order A > CD > S. These results indicate markedly different degrees of carbon consumption depending on the activating atmosphere. In particular, the substantially lower yield obtained for sample S demonstrates that steam promoted much more extensive gasification of the carbon matrix than either CO_2_ or air. Similar behaviour has been reported for the physical activation of tyre-derived chars, for which steam generally produces greater burn-off and more pronounced pore development than CO_2_ under comparable conditions [68,69,70,71].

The relatively high activation yields of A and CD suggest that gasification remained comparatively limited under the conditions employed. Tyre-derived chars possess a compact structure with initially restricted accessible porosity, which can hinder the penetration of activating gases towards internal reactive sites. These sites are mainly associated with structural defects, edge carbon atoms and oxygen-containing surface functionalities, which are recognised as important centres governing the gasification reactivity of carbonaceous materials [72,73]. Consequently, the accessibility of these sites can strongly influence the extent of carbon conversion during activation.

Although both steam and CO_2_ activation proceed through carbon gasification reactions, steam generally exhibits faster gasification kinetics under comparable conditions. The much lower yield of S relative to CD is therefore consistent with its greater burn-off. Progressive gasification can open previously inaccessible pores and widen pre-existing ones, promoting both micropore development and improved pore connectivity. This interpretation is consistent with the more extensive structural changes subsequently observed for S by N_2_ adsorption and mercury intrusion porosimetry, as described in the following section.

The activation yield nevertheless provides only an indirect measure of structural development. Moderate gasification may generate new micropores and open blocked porosity, whereas more extensive burn-off can progressively widen existing pores and favour mesopore formation and pore interconnectivity. Excessive gasification may ultimately lead to micropore widening or partial deterioration of the pore walls. The final textural properties therefore depend on the balance between carbon consumption and pore development. The markedly different activation yields obtained with air, CO_2_ and steam thus provide an initial indication that the activating atmosphere governs the extent of structural reorganisation of the tyre-derived char, an effect examined quantitatively in the following section.

### 3.2. Characterisation of the ACs

#### 3.2.1. Textural Characterisation

**Figure 1:**
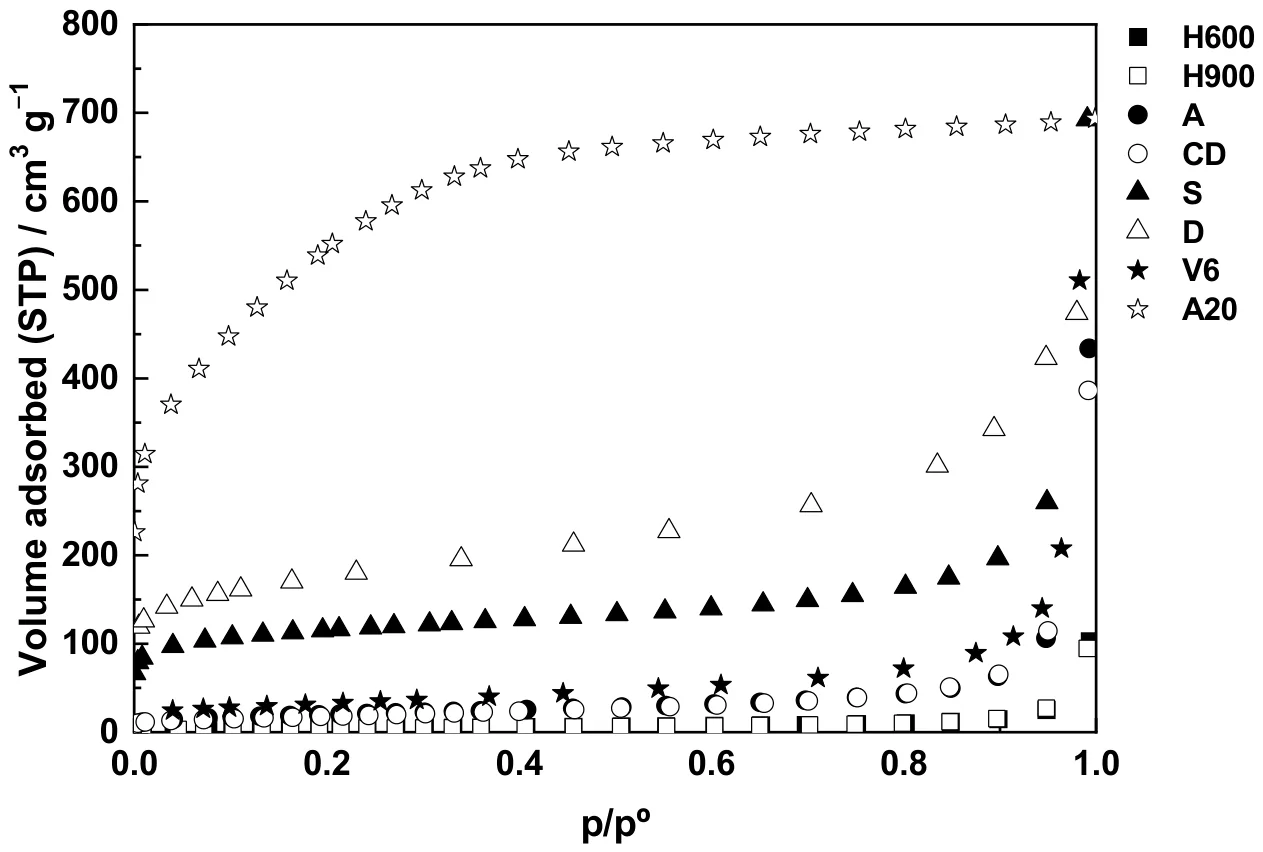
Adsorption isotherms of N_2_ at −196 °C.

The N_2_ adsorption isotherms measured at −196 °C for untreated UTR, the carbonised products (H600 and H900), the activated carbons (A, CD and S) and the commercial carbon materials (A20, D and V6) are presented in Figure 1.

As expected, UTR exhibits negligible nitrogen adsorption throughout the entire relative pressure (P/P_0_) range, indicating the virtual absence of accessible micro- and mesoporosity (Table 2). This behaviour reflects the compact structure of the original polymeric matrix, whose internal free volume is essentially inaccessible to nitrogen molecules.

Following pyrolysis, the carbonised products H600 and H900 exhibit a substantial increase in nitrogen adsorption. Their adsorption isotherms correspond to Type IV according to the IUPAC classification [74], indicating the presence of a significant mesoporous contribution. Indeed, both chars exhibit mesopore volumes of 0.14 and 0.16 cm^3^ g^−1^ for H600 and H900, respectively (Table 2). In addition, they contain a limited but significant amount of microporosity, with micropore volumes close to 0.05 cm^3^ g^−1^ according to the Dubinin–Radushkevich equation and approximately 0.03 cm^3^ g^−1^ when estimated directly from the adsorption isotherms (Table 2). These micropores most probably originate from the removal of large amounts of volatile low-molecular-weight compounds during pyrolysis, leaving behind an incipient pore network within the carbonaceous matrix. Thus, carbonisation primarily generates a carbonaceous precursor with only incipient accessible porosity, whereas the subsequent activation step determines the final extent and accessibility of the pore network.

The adsorption isotherms of samples A and CD remain remarkably similar to those of their corresponding precursor chars. Compared with H600 and H900, no significant changes are observed in either the shape of the isotherms or the amount of nitrogen adsorbed throughout the relative pressure range. Likewise, the quantitative textural parameters summarised in Table 2 reveal only marginal variations in BET surface area, micropore volume and mesopore volume. These results indicate that activation in air at 400 °C and in CO_2_ at 850 °C induced only limited gasification of the carbon framework under the experimental conditions employed. Consequently, the initially blocked pore system remained largely inaccessible, demonstrating that neither atmosphere was sufficiently effective to promote extensive pore opening or substantial micropore development. These observations are in agreement with the relatively high activation yields discussed in the previous section.

Steam activation produced a markedly different structural evolution. Sample S exhibits a pronounced increase in nitrogen adsorption at very low relative pressures, characteristic of extensive micropore development, followed by a continuous increase in adsorption over intermediate and high relative pressures, indicating the simultaneous presence of well-developed mesoporosity. Consequently, the adsorption isotherm combines the characteristics of Type I and Type IV behaviours, reflecting the formation of a hierarchical micro-/mesoporous network. Quantitative analysis confirms this remarkable structural evolution. Steam activation increases the BET surface area to 344 m^2^ g^−1^ while raising the micropore volume (W_0_) to 0.23 cm^3^ g^−1^. At the same time, mesoporosity and macroporosity are also enhanced, with V_me_, V_me-p_ and V_ma-p_ values higher than those obtained for the corresponding precursor char and for samples A and CD (Table 2). Compared with the other activation routes, steam therefore produces a much greater development of microporosity while preserving an extensive transport pore network.

The commercial carbon materials provide a valuable reference for interpreting the structural evolution of the prepared carbons. Carbon fibre A20 exhibits by far the highest nitrogen adsorption capacity, corresponding to a Type I isotherm with a particularly open knee, indicative of a highly developed microporous structure extending into the narrow mesopore region. Such porosity is characteristic of activated carbon fibres, where pores originate from the imperfect stacking of graphite microfibrils, generating numerous voids directly accessible from the external surface [75]. Commercial activated carbon D also exhibits a typical Type I isotherm, although its microporosity is composed predominantly of narrower pores, as generally observed for conventional activated carbons. In contrast, V6 displays adsorption behaviour characteristic of a predominantly mesoporous carbon.

#### 3.2.2. Surface Area and Microporosity

The textural parameters derived from the N_2_ adsorption isotherms using the BET and Dubinin–Radushkevich (D–R) methods are summarised in Table 2. The reported values include the BET surface area (S_BET_), the micropore volume obtained from the D–R equation (W_0_), the micropore volume estimated from nitrogen adsorption (V_mi_) and the mesopore volume (V_me_) also from N_2_ adsorption data. As expected, untreated UTR exhibits negligible S_BET_ and W_0_ values because of the virtual absence of accessible porosity, confirming the non-porous nature of the original rubber matrix.

The carbonised products H600 and H900 exhibit relatively low BET surface areas of 61 and 64 m^2^ g^−1^, respectively, together with micropore volumes (W_0_) of 0.05 cm^3^ g^−1^. These values are comparable to those previously reported for used tyre-derived chars, where S_BET_ between 18–47 and 78–85 m^2^ g^−1^ have been obtained depending on the pyrolysis conditions employed [15,27]. The agreement with the literature confirms that pyrolysis alone produces only limited development of accessible microporosity despite generating the incipient pore network described in the previous section.

Activation in air and CO_2_ produces only minor modifications of the textural properties. Compared with their corresponding precursor chars, samples A and CD exhibit only slight increases in S_BET_, W_0_ and V_mi_, confirming that these activation treatments were unable to substantially develop the internal porous structure under the experimental conditions employed. These findings further demonstrate that both activation atmospheres induced only limited gasification of the carbon framework, in agreement with the adsorption isotherms discussed above.

In contrast, steam activation produces a remarkable enhancement of both surface area and microporosity. Sample S reaches a BET surface area of 344 m^2^ g^−1^ together with a micropore volume (W_0_) of 0.23 cm^3^ g^−1^ and a V_mi_ value of 0.17 cm^3^ g^−1^. These values are substantially higher than those of H900, A and CD, clearly demonstrating the much greater effectiveness of steam in developing accessible microporosity. The BET surface area obtained for sample S lies within the wide range of 270–1317 m^2^ g^−1^ previously reported for steam-activated carbons prepared from UTR [76,77,78]. Such variability mainly reflects differences in activation temperature, residence time and burn-off. As expected, more severe activation conditions generally lead to greater burn-off and, consequently, to more extensive surface area development.

Comparison with the commercial carbon materials further highlights the effectiveness of steam activation. The BET surface area and micropore volume of sample S are considerably higher than those of carbon black V6 and only moderately lower than those of commercial activated carbon D. This comparison is particularly meaningful because V6 is essentially carbon black, D is a conventional activated carbon, whereas sample S originates from used tyre char composed predominantly of partially gasified carbon black that has acquired the characteristics of an activated carbon through steam activation. These findings demonstrate the effectiveness of the activation strategy employed in the present work.

As expected, activated carbon fibre A20 exhibits by far the highest degree of textural development, with a BET surface area of 1876 m^2^ g^−1^, a micropore volume (W_0_) of 0.97 cm^3^ g^−1^ and a V_mi_ value of 0.70 cm^3^ g^−1^. These exceptionally high values are consistent with the manufacturing process of activated carbon fibres. Preparation at relatively low temperatures generates a very large number of extremely small pores distributed throughout the carbon fibre, whereas higher preparation temperatures generally produce fewer but wider pores [75]. Consequently, A20 possesses a much more highly developed microporous structure than any of the other materials investigated.

Overall, the degree of development of surface area and microporosity follows the order: A20 ≫ D > S > V6 > A ≈ CD ≈ H900 ≈ H600. Across all the carbon materials investigated, S_BET_ varies between 61 and 1876 m^2^ g^−1^, W_0_ between 0.05 and 0.97 cm^3^ g^−1^, and V_mi_ between 0.03 and 0.70 cm^3^ g^−1^.

#### 3.2.3. Meso- and Macroporosity

**Figure 2:**
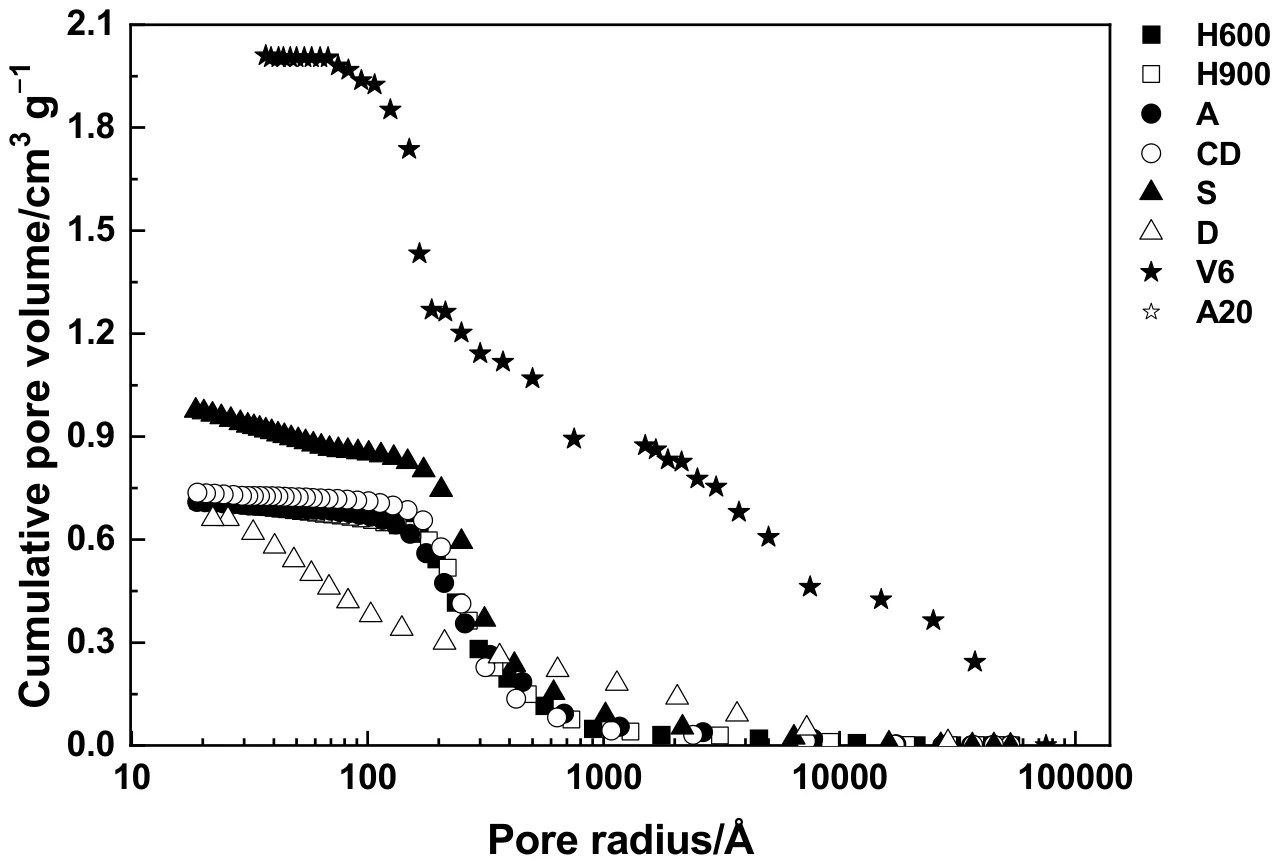
Curves of cumulative pore volume versus pore radius.

The development of meso- and macroporosity was further investigated by mercury intrusion porosimetry. In combination with nitrogen adsorption, this technique provides a comprehensive description of the porous structure by extending the characterisation towards the larger pore-size domain. The cumulative intrusion curves are presented in Figure 2, while the mesopore volume determined from nitrogen adsorption (V_me_) together with the meso- and macropore volumes obtained by mercury intrusion (V_me-p_ and V_ma-p_, respectively) are summarised in Table 2.

The mercury intrusion curves reveal clear differences in pore-size distribution among the prepared and commercial carbon materials. Samples S, D and A20 exhibit predominantly mesoporous structures, although their mesoporosity is clearly heterogeneous, particularly in the case of D and A20. In contrast, H600, H900, A and CD are characterised by pore systems composed mainly of wide mesopores and narrow macropores, reflecting the limited structural evolution produced by carbonisation and by activation in air or CO_2_. Carbon black V6 displays a distinct behaviour, exhibiting a relatively homogeneous pore-size distribution within the wide mesopore region but a heterogeneous macroporous domain characterised by a pronounced maximum centred at approximately 166 Å [79].

Comparison of the activated carbons with their corresponding precursor chars clearly demonstrates that substantial development of meso- and macroporosity occurs only after steam activation. Relative to H900, sample S exhibits noticeable increases in V_me_, V_me-p_ and V_ma-p_ (Table 2), confirming that steam activation promotes not only micropore generation but also extensive pore widening and improved pore interconnectivity. By contrast, activation in air and CO_2_ produces only minor changes in these parameters. The small variations observed in V_me-p_ and V_ma-p_ indicate that gasification occurred mainly at the walls of pre-existing macro- and mesopores, producing only limited modification of the overall pore network.

Considering all the pyrolytic chars and activated carbons, V_me-p_ ranges from 0.31 to 0.38 cm^3^ g^−1^, whereas V_ma-p_ varies between 0.37 and 0.59 cm^3^ g^−1^. These values indicate that the UTR-derived carbons are generally more macroporous than mesoporous when evaluated by mercury intrusion. Comparison with the commercial carbons further emphasises these differences. Relative to sample S, the degree of mesoporosity is similar for commercial activated carbon D but considerably higher for A20 and especially for V6. Macroporosity is highly developed in V6, decreases markedly in D and is almost absent in A20. It is also noteworthy that V6 exhibits a much lower mesopore volume determined by nitrogen adsorption (V_me_) than that obtained by mercury intrusion (V_me-p_). This discrepancy may arise from restricted accessibility of nitrogen molecules at −196 °C to part of the mesoporous network and/or from non-ideal effects associated with mercury intrusion, highlighting the complementary nature of both characterisation techniques.

**Figure 3:**
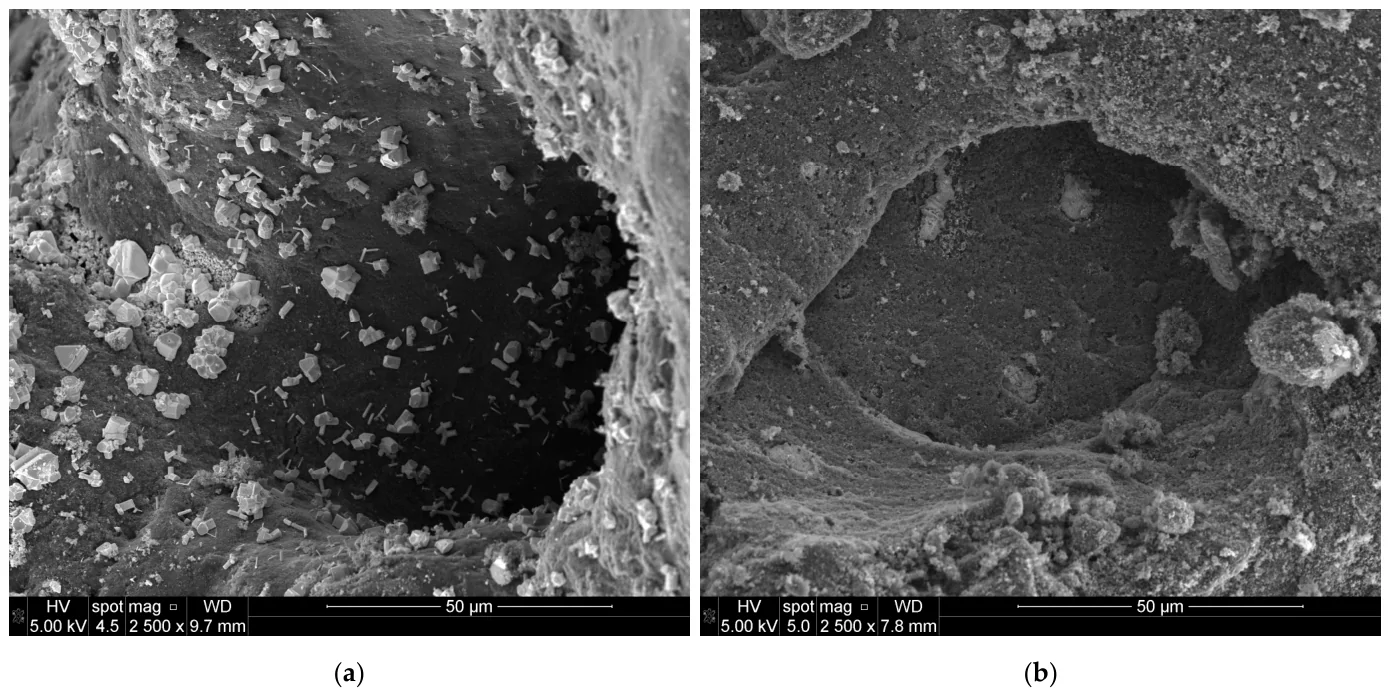
SEM micrograph (**a**) CD; (**b**) S.

Representative SEM micrographs of samples CD and S are shown in Figure 3 to illustrate the contrasting morphologies produced by CO_2_ and steam activation, respectively. In both materials, pores open to the external surface are clearly visible, confirming the porous nature of the activated carbons. Nevertheless, marked morphological differences are evident. Sample CD retains a comparatively compact carbon framework with relatively few surface openings, consistent with its limited textural development. In contrast, sample S exhibits a much more open and irregular morphology characterised by abundant cavities and interconnected voids generated during steam gasification. Although SEM cannot resolve the microporous domain responsible for most of the internal surface area, these observations provide direct visual evidence of the extensive structural reorganisation induced by steam activation and are fully supported by the nitrogen adsorption and mercury intrusion results.

#### 3.2.4. Surface Chemistry

Besides porous structure, the adsorption behaviour of carbonaceous materials is also influenced by surface chemistry, since the nature and abundance of surface functional groups determine the type and strength of the interactions established with adsorbate molecules. Consequently, Fourier-Transform Infrared Spectroscopy (FT-IR) and determination of the point of zero charge (pH_pzc_) were employed to evaluate the influence of the different activation atmospheres on the surface chemistry of the used tyre-derived carbons.

**Figure 4:**
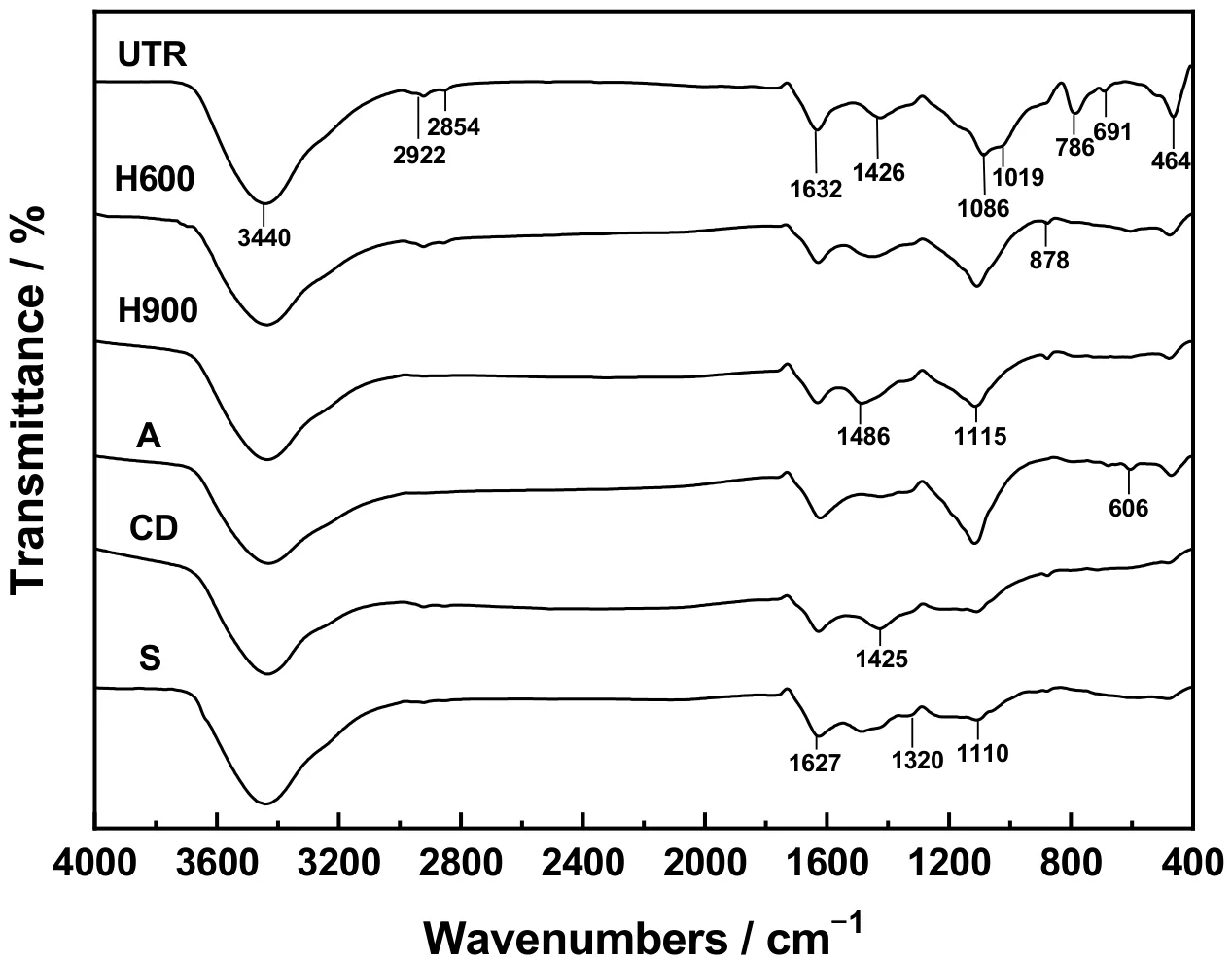
FT-IR spectra of the samples UTR, H600, H900, A, CD and S.

The FT-IR spectra recorded for UTR, H600, H900 and the corresponding activated carbons are presented in Figure 4. Overall, the spectra display a relatively small number of absorption bands, most of them of low intensity, reflecting the predominantly carbonaceous nature of the materials after pyrolysis and activation. Based on the expected chemical composition of UTR, the principal absorption bands have been tentatively assigned as summarised in Table 3 [75,78,79,80].

For untreated rubber, the broad absorption band centred at approximately 3440 cm^−1^ is attributed to the stretching vibration of hydroxyl groups associated with adsorbed water and oxygen-containing surface functionalities. Water retained within the rubber matrix may also contribute to the absorption observed around 3500 and 1650 cm^−1^. The band at 1426 cm^−1^ is assigned to aromatic C=C skeletal vibrations associated with the carbon black incorporated into the tyre formulation. In the 1310–1000 cm^−1^ region, infrared absorption arises mainly from C–O stretching vibrations of ether-type structures and hydroxyl-containing groups, although contributions from peroxide and hydroperoxide species through O–O stretching vibrations, as well as from inorganic components such as SiO_2_, cannot be excluded [81].

Comparison of the UTR spectra with those of H600 and H900 reveals that thermal treatment modifies both the organic and inorganic fractions of the original material. Following pyrolysis, the band at 1426 cm^−1^ decreases in intensity, whereas the absorption centred around 1115 cm^−1^ becomes more pronounced. Most of the remaining absorption bands either decrease progressively or disappear after heat treatment, indicating the elimination of oxygenated and aliphatic structures together with the progressive aromatisation of the carbon matrix. Among all the bands, those centred at approximately 3440 and 1632 cm^−1^ remain practically unchanged throughout the UTR-derived series, whereas the band at 1426 cm^−1^ is preserved in all samples except A.

Only minor spectral modifications are produced by activation in air and CO_2_. The FT-IR spectra of samples A and CD remain very similar to those of their corresponding precursor chars, apart from slight variations in the intensity of several absorption bands, particularly in the 1100 cm^−1^ region. These results indicate that physical activation under the conditions employed produces only limited changes in surface chemistry compared with the much more pronounced structural modifications discussed in the previous sections.

Steam activation produces somewhat more noticeable spectral changes. Sample S exhibits a larger number of better-defined absorption bands than the remaining UTR-derived carbons, although their overall intensities remain relatively low. This suggests that steam activation modifies the distribution of oxygen-containing surface functionalities to a greater extent than air or CO_2_ activation do. Nevertheless, the overall differences remain moderate, indicating that physical activation affects surface chemistry much less extensively than it modifies the porous structure of the carbon framework. These observations are consistent with previous studies showing that physical activation primarily promotes controlled gasification of the carbon matrix, whereas chemical activation generally induces much more extensive modification of surface functional groups [68,82].

**Figure 5:**
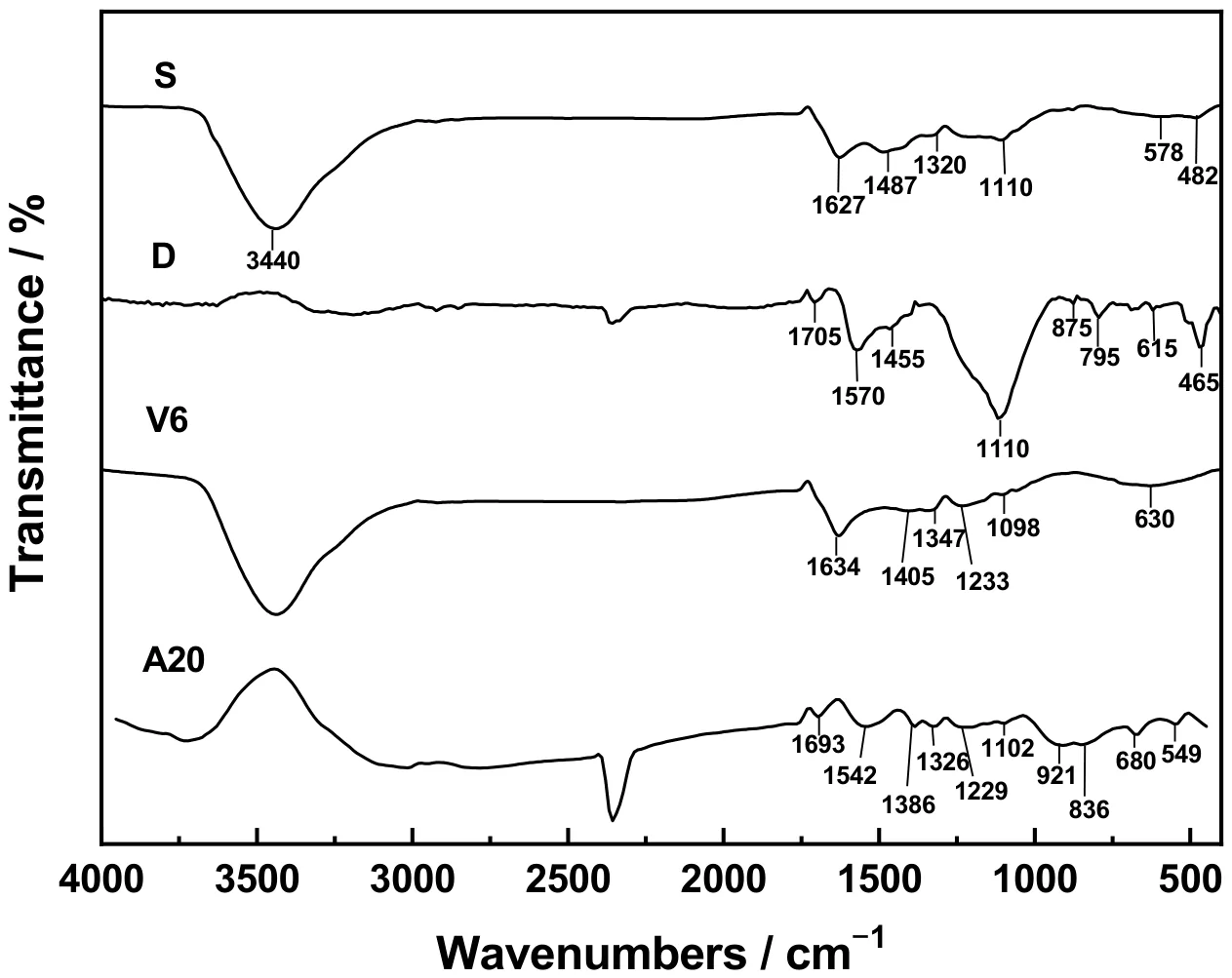
FT-IR spectra of the samples S, D, V6 and A20.

The FT-IR spectra of sample S together with those of the commercial carbon materials are presented in Figure 5. Overall, the spectra display a similar number of absorption bands, indicating that the different carbonaceous materials possess broadly comparable surface chemistries. Nevertheless, significant differences in the intensity of several bands, particularly within the 1600–600 cm^−1^ region, reveal variations in the concentration of oxygen-containing surface groups. It is also noteworthy that the spectrum of V6 closely resembles that of sample S. The absorption bands centred at approximately 1705, 1570, and 1110 cm^−1^ are compatible with C=O stretching vibrations in carboxylic acid groups or quinone-type structures, aromatic C=C skeletal vibrations and C–O–C stretching vibrations in ether structures, respectively. Activated carbon fibre A20 exhibits a larger number of weak absorption bands distributed throughout the 470–1750 cm^−1^ region, suggesting the presence of a greater variety of surface functionalities than in the remaining carbonaceous materials, although their overall concentration appears to be relatively low. This distinctive surface chemistry may be related to the broad pore-size distribution of A20, which facilitates oxygen access to reactive surface sites during manufacture and storage. In contrast, the broad absorption bands centred around 3440 and 1634 cm^−1^ are absent from the spectra of D and A20, indicating differences in the abundance of hydroxyl-containing surface species relative to the UTR-derived carbons.

The acid–base character of the carbon surfaces was hence evaluated by determining the point of zero charge (pH_pzc_), the corresponding values being summarised in Table 2. Most UTR-derived materials exhibit basic surfaces, with pH_pzc_ values ranging between approximately 9.0 and 10.4. The only exception is sample A, whose pH_pzc_ decreases to 6.7 following air activation. Among the commercial carbons, V6 exhibits a circumneutral surface (pH_pzc_ = 7.1), whereas D displays the most acidic character (pH_pzc_ = 3.6), with A20 presenting an intermediate value (pH_pzc_ = 5.8).

The observed variation (H900 > H600 and S > CD > A) demonstrates that both pyrolysis temperature and activation atmosphere influence the surface chemistry of the prepared carbons. However, these changes cannot be attributed solely to variations in the mineral fraction, since silica itself is an acidic oxide. Rather, the measured pH_pzc_ values most probably result from the combined effects of changes in mineral composition together with the formation, transformation and removal of oxygen-containing surface functional groups during pyrolysis and activation. In this context, the absence of the broad hydroxyl-related band around 3440 cm^−1^ in D and A20 is consistent with their comparatively lower pH_pzc_ values.

Although moderate differences exist in the abundance and distribution of oxygen-containing surface groups, the chemical characteristics of the UTR-derived carbons remain broadly similar according to the available FT-IR and pH_pzc_ data. The superior adsorption performance of the steam-activated carbon is consistent with its greater micropore development and improved pore accessibility; however, differences in surface chemistry may also contribute to the observed behaviour. Since the present characterisation does not allow the respective contributions of pore structure and surface chemistry to be quantitatively separated, no dominant role can be conclusively assigned to either factor.

It should be noted that the present study did not include a quantitative determination of individual acidic and basic surface groups (e.g., by Boehm titration) or element-specific surface analysis by XPS. Accordingly, the discussion of surface chemistry is restricted to the relative information provided by FT-IR and pH_pzc_ measurements. Overall, the FT-IR and pH_pzc_ results reveal moderate differences in surface functionality and acid–base character among the investigated carbon materials.

### 3.3. Adsorption of p-Chlorophenol (p-Cp)

#### 3.3.1. Adsorption Kinetics

**Figure 6:**
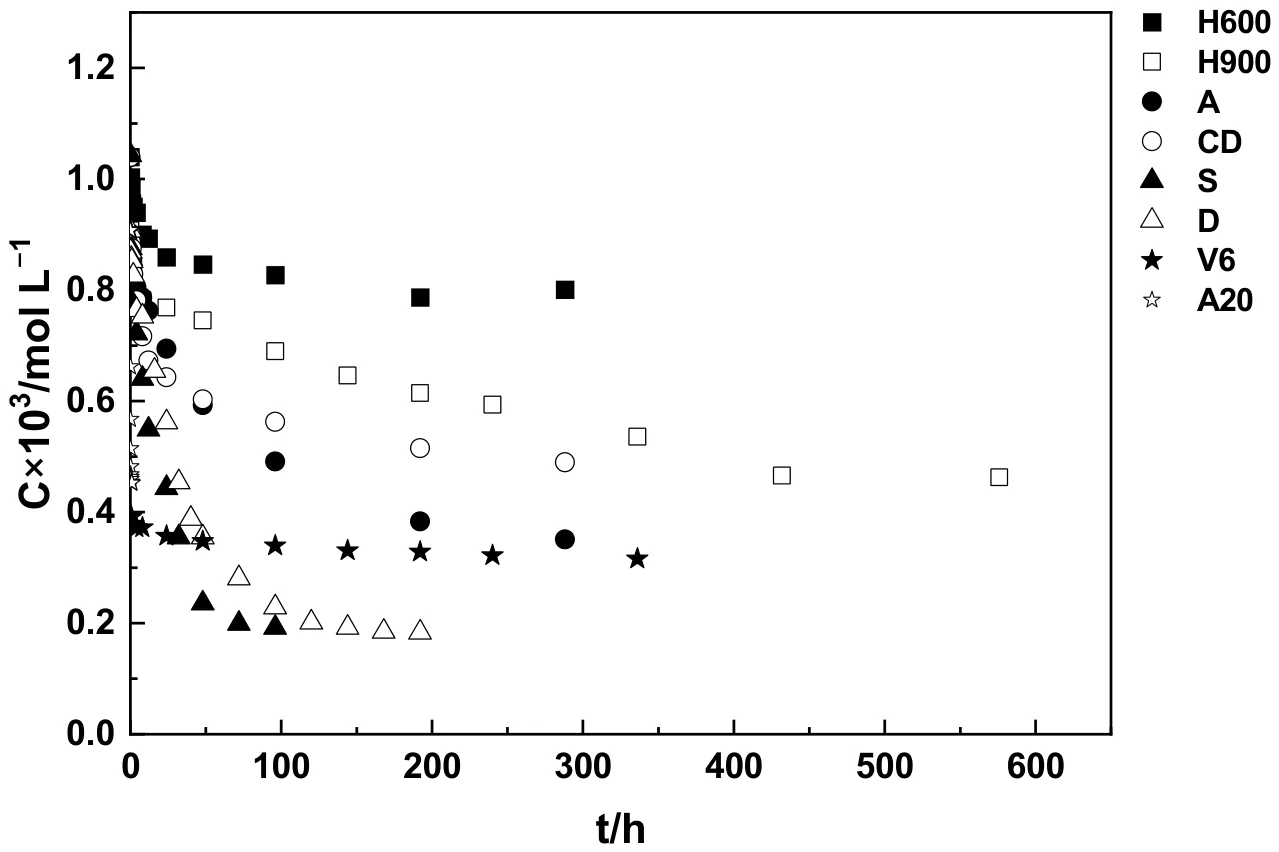
Adsorption kinetics of *p*-chlorophenol.

The adsorption kinetics of *p*-chlorophenol on the prepared and commercial carbon materials are presented in Figure 6, while the kinetic parameters obtained from the pseudo-first-order and pseudo-second-order models are summarised in Table 4.

All adsorption systems exhibit a similar overall kinetic profile characterised by a rapid initial uptake followed by a progressively slower approach to equilibrium. This is typical of adsorption on porous carbonaceous materials and reflects the progressive occupation of available adsorption sites together with the increasing diffusional resistance encountered as adsorption proceeds. During the initial stage, the large concentration gradient between the solution and the adsorbent surface provides a strong driving force for mass transfer, allowing rapid adsorption on the most accessible sites. As these sites become progressively occupied, adsorption increasingly depends on the transport of *p-C*p molecules towards less accessible regions within the porous network, resulting in a gradual decrease in adsorption rate.

Although the general kinetic behaviour is similar for all materials, important differences are observed in both adsorption rate and equilibrium uptake. Untreated UTR exhibits only negligible adsorption throughout the experimental period, confirming that the original rubber matrix lacks both the porous structure and the accessible adsorption sites required for efficient *p-C*p removal. The carbonised products H600 and H900 display only modest improvement, reaching relatively low adsorption capacities despite the limited pore development generated during pyrolysis.

Activation in air and CO_2_ produces only slight enhancements in adsorption performance. Samples A and CD exhibit somewhat higher adsorption capacities than their corresponding precursor chars, but both remain considerably less effective than the steam-activated carbon. This is consistent with the limited structural modifications produced by these activation treatments, which generated only minor increases in accessible surface area and microporosity.

In contrast, steam activation dramatically improves both adsorption rate and adsorption capacity. Sample S exhibits the steepest initial adsorption curve among the UTR-derived carbons and reaches equilibrium considerably faster than H600, H900, A and CD. Moreover, its equilibrium adsorption capacity is substantially higher, demonstrating that steam activation simultaneously enhances both the kinetics and the extent of adsorption. Comparison with the commercial carbon materials further illustrates the effectiveness of the steam activation process. As expected, activated carbon fibre A20 exhibits the fastest adsorption kinetics owing to its exceptionally high accessible surface area and highly developed microporous structure. Commercial activated carbon D also adsorbs *p-C*p rapidly, whereas carbon black V6 shows considerably slower adsorption despite possessing a relatively well-developed mesoporous structure.

The experimental kinetic data were fitted using the pseudo-first-order [58] and pseudo-second-order [59] models (Equations (2) and (3), respectively) over the interval from t = 0 to the experimentally determined equilibration time for each adsorption system. The fitted kinetic constants and equilibrium uptake values are reported in Table 4.

The relative performance of the two kinetic models depended on the adsorbent. The pseudo-second-order model provided very good fits for H600, CD, S, V6 and A20, whereas the pseudo-first-order model showed comparable or slightly higher correlation for A and D; H900 was described less satisfactorily by both equations. Accordingly, the kinetic models are used here as empirical descriptions of the adsorption-rate profiles and their statistical performance is not interpreted as evidence for a specific adsorption mechanism.

#### 3.3.2. Adsorption Equilibrium

**Figure 7:**
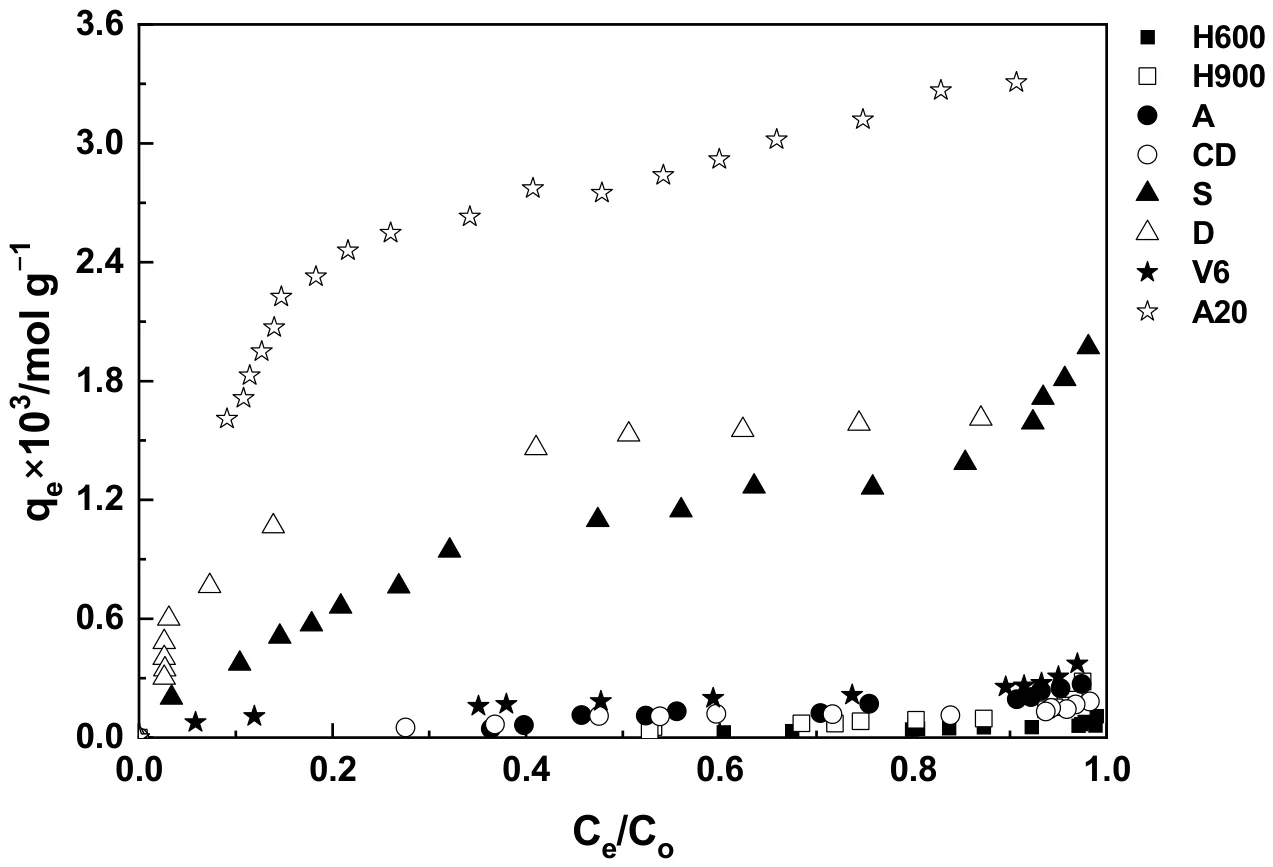
Adsorption isotherms of *p*-chlorophenol.

The adsorption isotherms of *p-C*p obtained for the prepared and commercial carbon materials are presented in Figure 7. Nonlinear fits to the Langmuir, Freundlich, Sips and Redlich–Peterson models are shown in Figure S1, and the complete fitting parameters and goodness-of-fit statistics are summarised in Table S1. Important differences are observed in both equilibrium uptake and isotherm shape depending on the porous characteristics of each adsorbent.

Untreated UTR exhibits negligible adsorption over the entire concentration range investigated, confirming that the original rubber matrix possesses neither the accessible porous structure nor the adsorption sites required for effective *p-C*p removal. Carbonisation produces only modest improvement. Samples H600 and H900 adsorb measurable amounts of *p-C*p, but their equilibrium capacities remain relatively low owing to their limited development of accessible microporosity. Activation in air and CO_2_ leads to only slight increases in adsorption capacity relative to the precursor chars. The adsorption isotherms of samples A and CD closely resemble those of H600 and H900, indicating that the limited structural modifications induced by these activation treatments are insufficient to produce a substantial increase in adsorption performance.

Steam activation, however, produces a marked improvement in adsorption performance. Sample S exhibits the highest equilibrium uptake among the UTR-derived carbons throughout the concentration range investigated. The corresponding isotherm rises steeply at low equilibrium concentrations, indicating strong adsorption, and continues to increase over the experimental range without a clearly defined saturation plateau.

Comparison of the equilibrium models revealed a strongly adsorbent-dependent behaviour (Table S1 and Figure S1). The additional flexibility provided by the Sips and Redlich–Peterson equations did not generally result in a substantially improved description of the experimental data. In several cases, these three-parameter models converged towards limiting forms already represented by Freundlich or Langmuir, whereas in others their parameters were poorly constrained. Consequently, model performance was interpreted considering not only goodness of fit but also model parsimony and parameter identifiability.

Clear differences in model performance were observed among the UTR-derived materials. For H600 and H900, the Freundlich equation provided the best overall description of the experimental data, although the fits were only moderate compared with those obtained for some of the more highly adsorbing materials. The nonlinear Freundlich exponents were greater than unity (1/*n* ≈2.55 and 3.55 for H600 and H900, respectively), and therefore should not be interpreted using the conventional criterion in which 0< 1/*n* <1 is associated with favourable adsorption. In both cases, the Sips model converged towards a Freundlich-like response without providing a sufficient improvement to compensate for the additional parameter. Conversely, the Langmuir and Redlich–Peterson equations tended towards essentially linear, Henry-type limiting behaviour, indicating that a physically meaningful saturation capacity could not be identified within the experimental concentration range. Consequently, Langmuir *Q_m_* and affinity parameters for these samples should not be assigned mechanistic significance.

Sample A showed a similar pattern. Freundlich gave the most favourable statistical description, with 1/*n* ≈ 1.39, whereas Sips reproduced practically the same superlinear concentration dependence and did not justify its additional parameter. Langmuir and Redlich–Peterson provided poorer fits and approached limiting forms close to linear behaviour. Thus, for H600, H900 and A, the additional flexibility of the three-parameter equations did not reveal a distinct equilibrium regime beyond that already shown by Freundlich. In contrast, CD exhibited no clear preference between the two conventional models. Langmuir and Freundlich gave almost identical fitting statistics, while Sips and Redlich–Peterson produced only negligible reductions in residual error and were penalised by *AICc*. The RP parameters also varied appreciably with the initial estimates while producing virtually unchanged fitted curves, indicating weak parameter identifiability. Accordingly, the equilibrium behaviour of CD cannot be uniquely assigned to either an ideal Langmuir-type or a purely Freundlich-type response.

For the steam-activated sample S, Freundlich provided the best balance between goodness of fit and model parsimony. Sips and Redlich–Peterson yielded residual errors very similar to that of Freundlich but converged towards Freundlich-like limiting behaviour, whereas their additional parameters were poorly constrained. Langmuir produced a somewhat poorer description of the data. Therefore, the high *p-C*p uptake exhibited by S among the UTR-derived carbons is better represented empirically by a heterogeneous power-law response over the concentration range investigated than by a uniquely defined monolayer saturation model. This result is consistent with the heterogeneous pore architecture developed by steam activation, although the mathematical fit alone cannot establish a specific adsorption mechanism.

The commercial materials showed three distinct behaviours. For activated carbon D, Langmuir clearly provided the preferred description. The Redlich–Peterson fit converged to *β* = 1, becoming mathematically equivalent to Langmuir, while the Sips exponent was also close to unity (*n_S_* ≈ 1.04). Thus, neither three-parameter model provided meaningful additional information, and the finite Langmuir capacity obtained for D can be considered well defined within the experimental range. In contrast, V6 was best described by Freundlich. Both Sips and Redlich–Peterson converged towards the Freundlich limiting form: for RP, 1 − *β* closely reproduced the Freundlich exponent and *K_R_/a_R_* closely reproduced *K_F_*. Their additional parameters therefore did not improve the description sufficiently to justify the increased complexity. Langmuir gave a poorer fit.

A20 differed from the remaining materials in that both Langmuir and Redlich–Peterson described the data very well. Redlich–Peterson yielded the lowest SSE and RMSE and the highest adjusted *R^2^*, whereas Langmuir gave the lowest *AICc*. The *AICc* difference between the two models was only about 1.1, indicating that they should be regarded as statistically competitive rather than as clearly distinguishable. The RP exponent (*β* ≈ 0.925) was close to the Langmuir limit, showing that the improvement introduced by the additional RP parameter was relatively small. Sips also produced a stable fit, with *n_S_* ≈ 0.862, suggesting a moderate departure from ideal Langmuir behaviour, but its higher *AICc* did not justify the additional parameter. Consequently, Langmuir remains the most parsimonious description of A20, with a well-defined fitted capacity of approximately 3.52 ×10^−3^ mol g^−1^, while RP provides a slightly better residual description.

Overall, the comparison of the four models demonstrates that no single isotherm equation describes all eight carbon materials equally well. Freundlich is preferred for H600, H900, A, S and V6; Langmuir and Freundlich are essentially indistinguishable for CD; Langmuir is clearly favoured for D; and Langmuir and Redlich–Peterson provide statistically comparable descriptions for A20. Importantly, the Sips and Redlich–Peterson equations generally converged towards limiting forms already represented by the simpler two-parameter models and rarely provided sufficient improvement to offset their additional complexity. The modelling therefore supports the use of the simplest statistically adequate equation for each adsorbent and cautions against assigning mechanistic significance to fitted parameters when saturation is not experimentally constrained.

#### 3.3.3. Relationship Between Porous Structure and Adsorption Performance

The combined textural and adsorption results indicate that the performance of the investigated carbons is governed by the overall organisation and accessibility of the porous network rather than by any single structural parameter. Although increasing BET surface area generally favours adsorption, the present results show that surface area alone cannot account for the differences observed among the materials. This is particularly evident when comparing sample S with the commercial carbons. Despite its substantially lower BET surface area than activated carbon D, the steam-activated carbon exhibits a markedly higher equilibrium uptake than the other UTR-derived materials and adsorption kinetics approaching those of the commercial adsorbents. Conversely, carbon black V6 possesses a relatively well-developed mesoporous structure but shows substantially lower adsorption performance than S. These observations demonstrate that neither total surface area nor total pore volume alone adequately describes the adsorption behaviour of the investigated materials.

The differences can be better understood by considering the complementary roles of the different pore-size domains. Micropores provide a large fraction of the adsorption space available to *p-C*p, whereas meso- and macropores primarily facilitate molecular transport towards the internal adsorption regions. Efficient adsorption therefore requires not only sufficiently developed microporosity but also an interconnected pore network that ensures accessibility to these adsorption sites. Samples H600, H900, A and CD illustrate the limitations associated with relatively poorly developed pore systems. Although these materials contain measurable microporosity, their equilibrium uptake and adsorption rates remain comparatively low. In contrast, steam activation promotes considerably more extensive gasification of the carbon matrix, simultaneously increasing micropore volume and developing wider transport pores. The resulting hierarchical micro-/mesoporous network therefore allows a larger fraction of the internal adsorption space to become accessible under the experimental conditions.

The equilibrium-model analysis is consistent with this diversity in porous architecture and further shows that the eight adsorbents do not share a single equilibrium response. Freundlich provided the most parsimonious description for H600, H900, A, S and V6, whereas D was clearly better represented by Langmuir. For CD, Langmuir and Freundlich were statistically almost indistinguishable, while for A20 the Langmuir and Redlich–Peterson equations provided comparably strong descriptions, with Redlich–Peterson yielding slightly lower residual errors but Langmuir retaining the lowest *AICc*. The additional Sips and Redlich–Peterson parameters generally did not produce sufficient improvement to justify the increased model complexity. In several cases, these equations converged towards limiting forms already represented by Freundlich or Langmuir, and some of their individual parameters were poorly constrained. The modelling results should therefore be regarded primarily as empirical descriptions of the different equilibrium responses rather than as evidence for distinct adsorption mechanisms.

This distinction is particularly relevant for the less porous UTR-derived materials. For H600 and H900, Langmuir and Redlich–Peterson tended towards essentially linear limiting behaviour, and no experimentally well-defined saturation capacity could be identified. Sample A showed a similar tendency, whereas its equilibrium data were better represented by the Freundlich equation. For S, both Sips and Redlich–Peterson approached Freundlich-like behaviour without improving the fit sufficiently to offset the additional parameter. The same tendency was observed for V6. Conversely, the convergence of Redlich–Peterson to its Langmuir limit for D, together with a Sips exponent close to unity, supports the suitability of the simpler Langmuir description for this material. A20 represents an intermediate case in which a slight departure from ideal Langmuir behaviour can be accommodated by the three-parameter models, although the improvement remains modest relative to their greater complexity.

Surface chemistry may also contribute to *p-C*p adsorption. The initial solution pH was approximately 5.0 and was not externally adjusted. Under these conditions, *p-C*p is expected to be overwhelmingly present in its neutral molecular form. At this pH, all the investigated carbon materials except D have pH_pzc_ values above the solution pH and are therefore expected to exhibit a net positive surface charge, whereas D (pH_pzc_ = 3.6) is expected to be negatively charged. Nevertheless, because *p-C*p is predominantly neutral under these conditions, electrostatic interactions are unlikely to dominate the adsorption process. The aromatic ring of *p-C*p may interact with the aromatic domains of the carbon matrix through π–π interactions, while dispersive forces and, depending on the surface functionalities present, hydrogen bonding may also contribute. However, pH was not systematically varied, and the available FT-IR and pH_pzc_ data do not allow the individual contributions of surface chemistry, surface charge and pore structure to be quantitatively separated. The influence of pH should therefore be regarded as a limitation of the present study.

The importance of pore accessibility is also evident from the kinetic results. Steam activation generates a more interconnected internal structure that reduces diffusional limitations and facilitates transport towards adsorption regions within the particles. The higher equilibrium uptake of S relative to H600, H900, A and CD therefore does not merely reflect an increase in micropore volume, but the development of microporosity that is more effectively connected to the external surface through a transport-pore network. Previous studies on activated carbons have similarly shown that adsorption performance depends on the combined effects of micropore development, pore accessibility, pore connectivity and molecular diffusion rather than on BET surface area alone [83,84,85,86,87]. Overall, steam activation produced the most favourable porous architecture among the UTR-derived materials investigated, combining increased micropore development with improved internal transport pathways. This structural evolution is consistent with both the higher experimental p-Cp uptake and the faster adsorption observed for S relative to the other UTR-derived carbons. At the same time, the comparison of the four equilibrium models demonstrates that the adsorption behaviour cannot be reduced to a single idealised isotherm or interpreted in terms of a unique adsorption mechanism. Instead, the results support a processing–structure–performance relationship in which activation atmosphere controls pore development and accessibility, which in turn strongly influence the adsorption response.

The present study, nevertheless, was designed as a comparative processing–structure–adsorption investigation rather than as a complete process-optimisation or scale-up study. Systematic optimisation of pH, temperature, initial concentration and adsorbent dosage was therefore outside its scope. Because the adsorption experiments were performed at a single temperature (25 °C), thermodynamic parameters were not calculated; temperature-dependent equilibrium measurements will be required for a rigorous thermodynamic analysis. Regeneration and repeated adsorption–desorption cycles were also not investigated and should be addressed before practical reuse can be assessed.

## 4. Conclusions

Used tyre rubber was successfully converted into carbonaceous adsorbents by pyrolysis followed by physical activation in air, CO_2_ or steam. The activation atmosphere was the key variable controlling pore development. Air and CO_2_ produced only minor structural modifications under the conditions investigated, whereas steam activation increased S_BET_ from 64 to 344 m^2^ g^−1^ and the D–R micropore volume from 0.05 to 0.23 cm^3^ g^−1^ while simultaneously developing a more extensive meso-/macroporous transport network.

The steam-activated carbon S exhibited the highest experimental *p-C*p uptake among the UTR-derived materials together with markedly improved adsorption kinetics. Equilibrium modelling showed that no single isotherm equation adequately described all eight carbon materials. Freundlich provided the most parsimonious description for H600, H900, A, S and V6; Langmuir was clearly preferred for D; Langmuir and Freundlich were essentially equivalent for CD; and Langmuir and Redlich–Peterson provided statistically comparable descriptions for A20. The Sips and Redlich–Peterson equations generally did not improve the fits sufficiently to justify their additional parameters and frequently converged towards limiting forms already represented by the simpler Freundlich or Langmuir equations. These results also demonstrate that fitted saturation capacities should only be interpreted when a saturation regime is adequately constrained by the experimental data.

Taken together, the textural, kinetic and equilibrium results indicate that adsorption performance is governed by the combined effects of accessible microporosity and pore-network connectivity rather than by BET surface area alone. The available FT-IR and pH_pzc_ data indicate only moderate differences in surface chemistry among the UTR-derived carbons, although its contribution cannot be quantitatively separated from that of pore structure. Steam activation therefore provided the most effective route investigated here for transforming UTR char into an efficient *p-C*p adsorbent by generating a hierarchical porous structure that combines adsorption space with efficient internal mass transport.

The present study establishes a processing–structure–performance framework for the valorisation of used tyre rubber as a precursor of carbonaceous adsorbents. Regeneration and repeated adsorption–desorption cycles, temperature-dependent adsorption, process optimisation and scale-up remain to be investigated. In addition, a complete assessment of environmental performance will require a dedicated life-cycle analysis based on process-scale material and energy inventories.

## Figures and Tables

**Table 1:** Preparation conditions, yields, and sample codes of the UTR-derived carbonaceous adsorbents.

**Subs**	**MHTT/°C**	**St/h**	**Atmosphere/AA**	**Yield/wt.%**	**Codes**
UTR	600	2	N_2_	36.6	H600
UTR	900	2	N_2_	33.0	H900
H600	400	2	Air	95.0	A
H900	850	2	CO_2_	88.0	CD
H900	850	2	Steam	54.0	S

Abbreviations: Subs, substratum; MHTT, maximum heat treatment temperature; St, soaking time; AA, activating agent.

**Table 2:** Characterisation of the samples.

**Sample**	**^1^ S_BET_** **/m^2^ g^−1^**	**^1^ W_0_** **/cm^3^ g^−1^**	**^1^ V_mi_** **/cm^3^ g^−1^**	**^1^ V_me_** **/cm^3^ g^−1^**	**^2^ V_me-p_** **/cm^3^ g^−1^**	**^2^ V_ma-p_** **/cm^3^ g^−1^**	**pH_pzc_**
UTR	0	0.001	0.00	0.001	-	-	6.7
H600	61	0.05	0.03	0.14	0.31	0.42	9.0
H900	64	0.05	0.03	0.16	0.35	0.37	10.4
A	68	0.05	0.03	0.14	0.36	0.37	6.7
CD	64	0.05	0.03	0.15	0.32	0.41	9.2
S	344	0.23	0.17	0.24	0.38	0.59	10.2
V6	115	0.05	0.04	0.21	0.75	1.26	7.1
D	618	0.26	0.25	0.41	0.38	0.28	3.6
A20	1876	0.97	0.70	0.38	0.48	0.01	5.8

^1^ N_2_ adsorption isotherm: S_BET_, BET surface area; W_0_ micropore volume (Dubinin–Radushkevich equation); V_mi_, micropore volume (V_ad_ at p/p^0^ = 0.10, V_ad_ = volume adsorbed); V_me_, mesopore volume (V_ad_ at p/p^0^ = 0.95 at p/p^0^ = 0.10). W_0_, V_mi_ and V_me_ are expressed as liquid volumes. ^2^ Mercury porosimetry: V_me-p_, mesopore volume; V_ma-p_, macropore volume.

**Table 3:** FT-IR band assignments for UTR.

**Band Position (cm^−1^)**	Bond Vibration *	**Atomic Grouping**	**UTR Component**
3440	ν (O-H)	-OH (-OH, H_2_O)	Carbon black, SiO_2_, ZnO
2922	ν_a_ (C-H)	-CH_2_-, -CH_3_	Rubber polymers
2854	ν_s_ (C-H)	-CH_2_-	Rubber polymers
1632	ν (C=O)	Quinone	Carbon black
	ν (C=C)	Aromatic ring	Carbon black
		Olefinic chain	Rubber polymers
	δ (O-H)	H_2_O	Carbon black, SiO_2_, ZnO
1426	δ (C-H)	CH2-, CH2=CH-	Rubber polymers
1426–1300 (shoulder)	δ (C-H)	Methyne groups	Rubber polymers
1300–1100 (shoulder)	ν (C-O)	-OH	Carbon black
1086	ν_as_ (C-O-C)	Ether structures	Carbon black
	ν (Si-O)		SiO_2_
	ν (Zn-O)		ZnO
1019	ν_s_ (C-O-C)	Ether structures	Carbon black
800–400	ν (Si-O)		SiO_2_
	γ (C-H)	Aromatic rings	Carbon black
	ν (C-S)	-C-Sn-C-	Vulcanized rubber
	ν (S-S)	-C-Sn-C-	Vulcanized rubber
	δ (O-H)	(-OH, H_2_O)	Carbon black, SiO_2_, ZnO
	δ (Si-O)		SiO_2_
	ν (Zn-O)		ZnO

* Symbols: ν, stretching; δ, bending (in-plane); γ, bending (out-of-plane); as, asymmetrical; s, symmetrical.

**Table 4:** Pseudo-first-order and pseudo-second-order fitting parameters for *p-C*p adsorption.

	**Pseudo-First Order Kinetic** **Model**			**Pseudo-Second Order Kinetic** **Model**		
**Sample**	**q_e_ × 10^6^** **(mol g^−1^)**	**k_1_** **(h^−1^)**	**R^2^**	**q_e_ × 10^6^** **(mol g^−1^)**	**k_2_** **(g mol^−1^ h^−1^)**	**R^2^**
H600	31.43	0.018	0.943	49.44	3551	0.996
H900	116.28	0.009	0.872	111.72	187	0.973
A	103.47	0.018	0.995	140.32	445	0.983
CD	70.11	0.017	0.987	110.42	1057	0.996
S	227.25	0.027	0.997	346.46	466	0.999
D	893.92	0.030	0.998	1007.49	49	0.992
V6	15.27	0.012	0.983	147.17	5302	1.000
A20	525.17	8.898	0.964	2290.18	67,675	1.000

## Data Availability

The original contributions presented in this study are included in the article. Further inquiries can be directed to the corresponding authors.

## Supplementary Materials

The following supporting information can be downloaded at: https://www.mdpi.com/article/10.3390/polym18202544/s1, Figure S1: Experimental equilibrium adsorption isotherms of *p*-chlorophenol and corresponding nonlinear fits to the Langmuir, Freundlich, Sips, and Redlich–Peterson models for samples; Table S1: Nonlinear isotherm-fitting parameters and goodness-of-fit statistics for *p*-chlorophenol adsorption on the investigated carbon materials.

## Abbreviations

The following abbreviations are used in this manuscript:

A	CAs developed by treatment of H600 at 400 °C in air
A20	Commercial carbon fibre
ACs	Activated carbons
BET	Brunauer–Emmett–Teller equation
CAs	Carbonaceous adsorbents
CD	CAs developed by treatment of H900 at 850 °C in carbon dioxide
D	Commercial activated carbon
D-R	Dubinin–Radushkevich
H600	CAs developed by heat treatment of UTR at 600 °C in N_2_
H900	CAs developed by heat treatment of UTR at 900 °C in N_2_
MHTT	Maximum heat treatment temperature
*p-C*p	*p*-chlorophenol
pH_pzc_	pH at the point of zero charge
S	CAs developed by treatment of H900 at 850 °C in steam
S_BET_	BET surface area
UTR	Used tyre rubber
V6	Commercial carbon black
W_0_	Micropore volume (Dubinin–Radushkevich equation)
