# Supplementary material for: Upcycling Used Tyre Rubber into Carbon Adsorbents: Effect of Physical Activation on Porous Structure and Comparative Evaluation for p-Chlorophenol Removal
Source: Polymers (Basel). 2026 Oct 16;18(20):2544. doi: 10.3390/polym18202544 (PMC13709235; doi:10.3390/polym18202544)
Supplement: Supplementary file 1 [file polymers-18-02544-s001.zip › polymers-4515857-supplementary.pdf]

Supplementary Materials

for

Upcycling Used Tyre Rubber into Carbon Adsorbents: Effect of Physical Activation on Porous Structure and Comparative Evaluation for *p*-Chlorophenol Removal

César Troca-Torrado, María Francisca Alexandre-Franco *, Juan Manuel Garrido-Zoido *, Eduardo Manuel Cuerda-Correa and Vicente Gómez-Serrano

Department of Organic and Inorganic Chemistry, Faculty of Science, University of Extremadura, Badajoz 06006, Spain; cesar_trocado@hotmail.com (C.T.-T); emcc@unex.es (E.M.C.-C.); vgomez@unex.es (V.G.-S.)
* Correspondence: malexandre@unex.es (M.F.A.-F.); jmgarridoz@unex.es (J.M.G.-Z.)

Figures: 1

Tables: 1

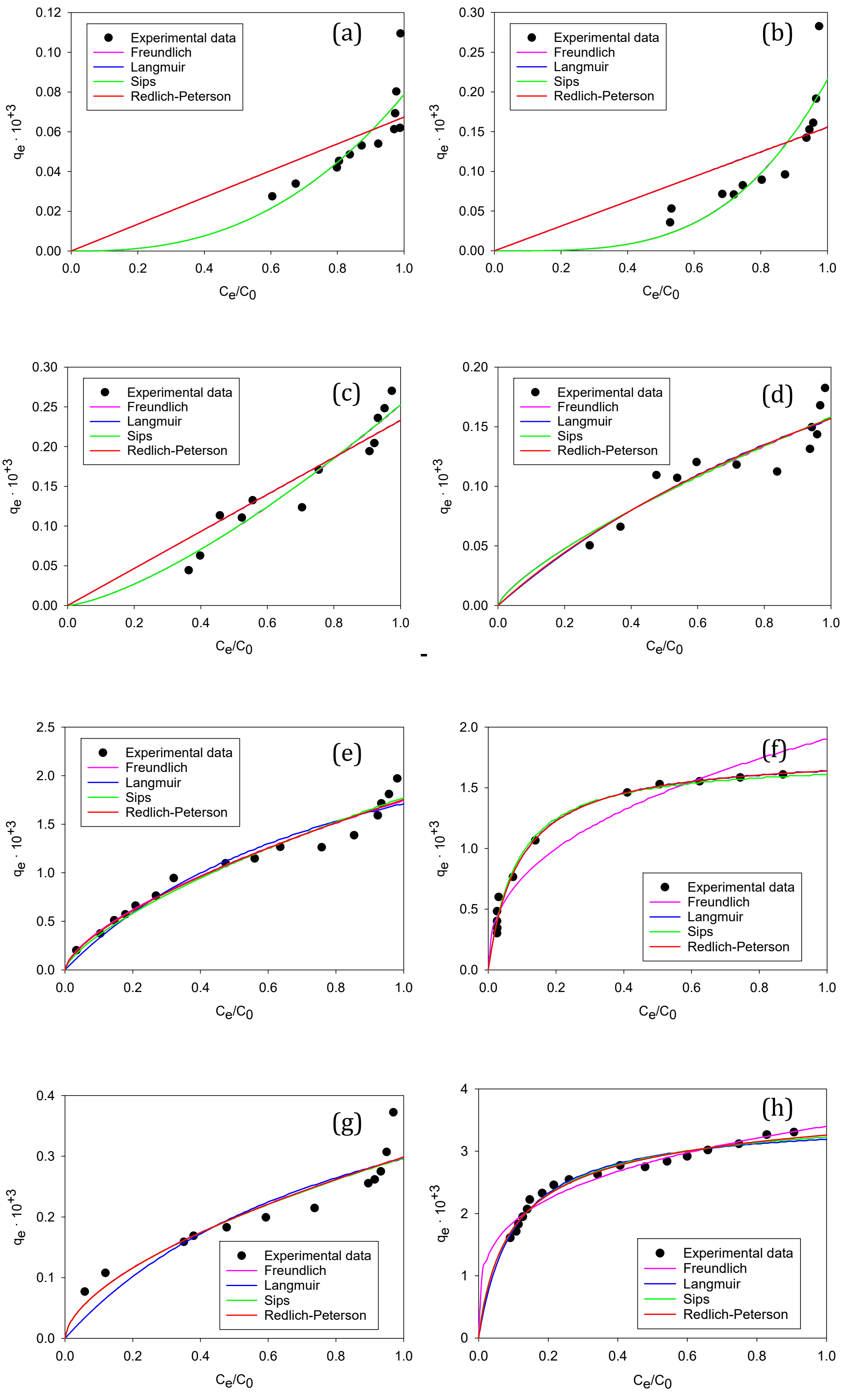

Figure S1. Experimental equilibrium adsorption isotherms of p-chlorophenol and corresponding nonlinear fits to the Langmuir, Freundlich, Sips, and Redlich–Peterson models for samples H600 (a), H900 (b), A (c), CD (d), S (e), D (f), V6 (g), and A20 (h). Where only two fitted curves are visually distinguishable, the Sips and Freundlich predictions are essentially coincident, as are those obtained from the Redlich–Peterson and Langmuir models.

Table S1. Nonlinear isotherm-fitting parameters and goodness-of-fit statistics for *p*-chlorophenol adsorption on the investigated carbon materials.

Sample	Model	Fitted parameters	SSE $(\text{mol g}^{-1})^2$	R^2	Adjusted R^2	RMSE (mol g^{-1})	AICc
H600	**Freundlich**	$K_F = 3.446\times10^3$; $1/n = 2.5470$	1.65069×10^{-9}	0.6917	0.6232	1.285×10^{-5}	**−267.15**
	Langmuir	Q_m, b: n.i.; $P = Q_m\ b = 6.74\times10^{-2}$ L g^{-1} (b→0)	2.78269×10^{-9}	0.4802	0.3647	1.668×10^{-5}	−260.88
	Sips	$q_S = 8.58\times10^{-3}$; $K_S = 159.994$; $n_S = 2.5552$	1.65×10^{-9}	0.6914	0.5757	1.35×10^{-5}	−263.47
	Redlich–Peterson	$K_R = 67.597$; $a_R = 1001.921$; $\beta \approx 0$	2.78269×10^{-9}	0.4802	0.2853	1.76×10^{-5}	−257.22
H900	**Freundlich**	$K_F = 9.58\times10^6$; $1/n = 3.5492$	1.21586×10^{-8}	0.7755	0.7257	3.487×10^{-5}	**−243.19**
	Langmuir	Q_m,, b: n.i.; $P = Q_m$, b = 0.1555 L g^{-1} (b→0)	2.89993×10^{-8}	0.4646	0.3456	5.385×10^{-5}	−232.76
	Sips	$q_S = 2.98\times10^{-1}$; $K_S = 131.975$; $n_S = 3.5695$	1.22×10^{-8}	0.7755	0.6913	3.68×10^{-5}	−239.52
	Redlich–Peterson	$K_R = 1522.814$; $a_R = 9791.531$; $\beta \approx 0$	2.89993×10^{-8}	0.4646	0.2639	5.68×10^{-5}	−229.09
A	**Freundlich**	$K_F = 3.69$; $1/n = 1.38845$	4.68449×10^{-9}	0.9199	0.9021	2.164×10^{-5}	**−254.63**
	Langmuir	Q_m,, b: n.i.; $P = Q_m$, b = 0.232873 L g^{-1} (b→0)	7.69226×10^{-9}	0.8685	0.8393	2.773×10^{-5}	−248.68
	Sips	$q_S = 1.96\times10^{-2}$; $K_S = 45.0454$; $n_S = 1.39966$	4.69×10^{-9}	0.9198	0.8897	2.28×10^{-5}	−250.95
	Redlich–Peterson	$K_R = 233.423$; $a_R = 1001.361$; $\beta \approx 0$	7.69226×10^{-9}	0.8685	0.8192	2.92×10^{-5}	−245.02
CD	Freundlich	$K_F = 2.69\times10^{-2}$; $1/n = 0.7439$	2.8138×10^{-9}	0.8223	0.7828	1.677×10^{-5}	−260.75
	**Langmuir**	Q_m, = 4.37×10^{-4} mol g^{-1}; b = 558.163 L mol^{-1}	2.7904×10^{-9}	0.8237	0.7846	1.670×10^{-5}	**−260.85**
	Sips	$q_S = 6.10\times10^{-3}$; $K_S = 8.34508$; $n_S = 0.75798$	2.81×10^{-9}	0.8224	0.7558	1.77×10^{-5}	−257.09
	Redlich–Peterson	$K_R = 0.26052$; $a_R = 226.240$; $\beta = 0.84478$	2.78879×10^{-9}	0.8238	0.7578	1.76×10^{-5}	−257.19
S	**Freundlich**	$K_F = 0.15025$; $1/n = 0.64492$	1.72894×10^{-7}	0.9599	0.9537	1.111×10^{-4}	**−288.57**
	Langmuir	Q_m, = 3.277×10^{-3} mol g^{-1}; b = 1.094×10^3 L mol^{-1}	2.2924×10^{-7}	0.9468	0.9386	1.280×10^{-4}	−284.05
	Sips	$q_S = 4.8567\times10^{-2}$; $K_S = 9.82104$; $n_S = 0.70782$	1.82×10^{-7}	0.9577	0.9472	1.183×10^{-4}	−284.67
	Redlich–Peterson	$K_R = 992.338$; $a_R = 6045.874$; $\beta = 0.34321$	1.72728×10^{-7}	0.9599	0.9499	1.153×10^{-4}	−285.51
D	Freundlich	$K_F = 3.0399\times10^{-2}$; $1/n = 0.40113$	1.71987×10^{-7}	0.9474	0.9357	1.311×10^{-4}	−211.40
	**Langmuir**	Q_m, = 1.789×10^{-3} mol g^{-1}; b = 1.090×10^4 L mol^{-1}	4.38057×10^{-8}	0.9866	0.9836	6.62×10^{-5}	**−227.81**
	Sips	$q_S = 1.726\times10^{-3}$; $K_S = 1.2420\times10^4$; $n_S = 1.04386$	4.58×10^{-8}	0.9860	0.9808	7.13×10^{-5}	−223.61
	Redlich–Peterson	$K_R = 19.5$; $a_R = 1.090\times10^4$; $\beta = 1.000$	4.38057×10^{-8}	0.9866	0.9816	6.98×10^{-5}	−224.14
V6	**Freundlich**	$K_F = 1.72\times10^{-2}$; $1/n = 0.58681$	1.04321×10^{-8}	0.8658	0.8360	3.230×10^{-5}	**−245.03**
	Langmuir	Q_m, = 5.66×10^{-4} mol g^{-1}; b = 1095.72 L mol^{-1}	1.38794×10^{-8}	0.8215	0.7818	3.726×10^{-5}	−241.60
	Sips	$q_S = 1.60\times10^{-2}$; $K_S = 1.23207$; $n_S = 0.59223$	1.05×10^{-8}	0.8651	0.8145	3.41×10^{-5}	−241.30
	Redlich–Peterson	$K_R = 146.088$; $a_R = 8515.495$; $\beta = 0.41372$	1.04399×10^{-8}	0.8657	0.8154	3.41×10^{-5}	−241.35
A20	Freundlich	$K_F = 2.08\times10^{-2}$; $1/n = 0.26207$	2.19278×10^{-7}	0.9542	0.9481	1.171×10^{-4}	−323.22
	**Langmuir**	Q_m, = 3.52×10^{-3} mol g^{-1}; b = 9745.61 L mol^{-1}	1.39536×10^{-7}	0.9709	0.9670	9.339×10^{-5}	**−331.36**
	Sips	$q_S = 3.72\times10^{-3}$; $K_S = 8830.10$; $n_S = 0.86199$	1.36×10^{-7}	0.9717	0.9656	9.51×10^{-5}	−328.95
	Redlich–Peterson	$K_R = 43.8170$; $a_R = 7418.357$; $\beta = 0.92513$	1.25877×10^{-7}	0.9737	0.9681	9.16×10^{-5}	−330.30

All parameters were obtained by unweighted nonlinear least-squares regression of the untransformed q_e *vs* C_e data.

Parameter units: Q_m and q_S, mol g^{-1}; b and K_S, L mol^{-1}; K_F, mol g^{-1} $(mol\ L^{-1})^{-1/n}$; K_R and P (= Q_m b), L g^{-1}; a_R, $(L\ mol^{-1})^{\beta}$; 1/n, n_S, and β are dimensionless. SSE is expressed in $(mol\ g^{-1})^2$, whereas RMSE is expressed in mol g^{-1}.n.i., not independently identifiable. For H600, H900 and A, Langmuir converged to a Henry-type limit (b→0, Qm→∞), so only P = Qmb is reported. Boundary or limiting Sips/RP solutions should be interpreted as empirical descriptions rather than as physically unique parameter sets.

Bold type identifies the lowest AICc for each sample. For CD, Langmuir and Freundlich are effectively equivalent (ΔAICc ≈ 0.10); for A20, Langmuir and Redlich–Peterson are statistically competitive (ΔAICc ≈ 1.06).
